# Genotypic Diversity of Human Papillomavirus (HPV) Types and Its Prevalence With Cervical Cancer (CC) in Central India

**DOI:** 10.7759/cureus.35227

**Published:** 2023-02-20

**Authors:** Sayali P Kulkarni, Shruti Paliwal, Susmit Kosta

**Affiliations:** 1 Obstetrics and Gynaecology, Sri Aurobindo Institute of Medical Sciences, Indore, IND; 2 Molecular and Virology Research Diagnostic Lab, Sri Aurobindo Institute of Medical Sciences, Indore, IND

**Keywords:** human papillomavirus genotypes, polymerase chain reaction, visual inspection with acetic acid (via), human papillomavirus (hpv), cervical cancer

## Abstract

Background

The high-risk human papillomavirus (hr-HPV) is linked with cervical cancer (CC), and the distinct proportional impact of each genotype on the prevalence of the disease depends on the area. Therefore, to find out the prevalence of HPV types in women with cervical lesions from central India, the current study was performed.

Methodology

Age, prior history of cervical disease, changes in lifestyle characteristics, menopausal status, and HPV vaccination status were all carefully gathered at enrollment for the 736 women (aged 21 to 60) screened in this cross-sectional study who were referred for regular screening of cervical during the study period. Cervix was examined for lesions by visual inspection with acetic acid (VIA) screening and HPV genotypes were identified by real-time polymerase chain reaction (RT-PCR).

Result

Among 736 women 215 (29.2%) were in the 21-30 age group, 321 (43.6%) in the 31-40 age group, 132 (17.9%) in the 41-50 age group, and 68 (9.3%) cases in >50 age group. According to education, there were 398 (54.1 %) with primary and below education, 115 (15.6%) with secondary education, and 223 (30.3%) with college and above education. HPV-16, 18, 31, and 45 each had a prevalence of 29.6%, 11.1%, 12.9%, and 9.2%, respectively, while the overall prevalence of hr-HP) was present in populations at 7.3% in individuals and 37.0% in combinations. Hr-HPV infection and prevalence were provocatively more (79.6%) in the VIA-positivity rate with CC.

Conclusion

Individual hr-HPV genotype prevalence was shown to be lower than with combinations (HPV-16, 18, 31, and 45). The HPV-16 genotype was identified to have a higher prevalence than HPV-18, 31, and 45. However, more awareness programs are needed for a better understanding of CC and HPV testing in central India.

## Introduction

High-risk human papillomavirus (hr-HPV) is assumed to be the only known cause of cervical cancer (CC) [1]. CC is the fourth most common and the leading cause of mortality in women worldwide, with 604,127 new cases and 341,831 fatalities every year. With 123,907 new cases and 77,348 fatalities per year, it is the second-most common cancer in India [2]. With low positive predictive value, since few women with any of the 12 types of HPV that are linked to cancer are present, hr-HPV will have an adhering disease that is more likely to progress to intrusive cancer [3]. The 16 and 18 genotypes of HPV are considered to be the most deeply involved in the development of CC, and they account for 60% of all cases [4-6]. 31, 33, 35, 45, 52, and 58 types of HPV are responsible for an extra 20% of CC cases [7]. A powerful indicator of the probability of neoplastic progression is one's HPV genotype [8]. It has been established that HPV testing offers advantages over conventional cytological examination in terms of higher output, greater sensitivity, and improved outcomes as a primary CC screening approach [9-11].

The World Health Organization (WHO) advises examination of HPV as the greatest CC prevention measure, particularly in low- and middle-income nations [12]. Visual inspection with acetic acid (VIA) detection and management of CC is still a crucial component of effective CC preventive and control programs, despite its limitations in terms of sensitivity and specificity. Studies have shown that VIA has a sensitivity range of 67% to 79% and a specificity range of 83% to 84% [13,14]. Therefore, the optimum strategy for a better result would be monitoring with VIA in conjunction with HPV tests.

The frequency of HPV transmission and cervical lesions among women of different ages is yet unknown. There is an urgent need for geographical screening for molecular-epidemiologic studies in regions that have verified the strong association between persistent infection by specific oncogenic-type HPVs and its relation with CC [15]. In various age groups that attended for routine CC screening, the diagnostic tests were performed independently or in combination for HPV-16, 18, 31, and 45 genotyping. This study's objective was to assess the prevalence of hr-HPV transmission and the VIA positivity rate in females who were at greater risk of contracting HPV.

## Materials and methods

It was conducted between September 2021 and August 2022 as a cross-sectional research. The study was performed at the Molecular Virology Research and Diagnostic Lab (MVRDL) and the Department of Obstetrics and Gynaecology at Sri Aurobindo Medical College & PG Institute, Sri Aurobindo University, Indore, India, between September 2021 and August 2022, including women willing to participate and who gave consent. Permission by the institutional ethical committee (IEC) Sri Aurobindo Institute of Medical Sciences, Indore, India with IEC number SAIMS/IEC/2021/16 was granted.

This study included a total of 736 women, ranging between 21 to 60 years, who were recommended for routine CC screening during the study period. At enrollment, thorough data were gathered through a self-administered questionnaire form (age, detailed history, menopausal status, HPV vaccination status, previous history of cervical disease, and changes in lifestyle characteristics). Women got a general and systemic examination after being interviewed and given their consent to participate in the study. A local examination of the vulva followed this to check for ulcerations, lesions, condylomata, redness, discharge, excoriation, and swelling. A speculum examination was made for abnormal bleeding from the cervix, ulcers, nabothian cysts, ectopy, cervicitis, warts, leukoplakia, polyps, and small papules on the cervix. Dimensions and form of the cervix. A vaginal examination for vaginitis or mucopurulent discharge was done.

Visual inspection of the cervix was performed using the following steps. The cervix was wiped of discharge, blood, or mucus using a dry cotton swab. The transformation zone, the red columnar epithelium, the squamocolumnar junction, the pink squamous epithelium, the red columnar epithelium, the anterior and posterior cervix lip, and all four fornices were recognized. Abnormal visual findings were noted at this moment. Applying 3% to 5% acetic acid to the cervix with a clean swab and timing the process for one minute allowed the acid to be absorbed. After one minute, the conversion zone was carefully examined for aceto white patches in the epithelium, paying particular attention to the area next to the squamocolumnar junction. The whiteness intensity, extent, location, borders, and demarcations and size of the aceto-white patches were noted. Swab devices were inserted into the endocervix and rotated three times counterclockwise to collect an endocervical swab sample. Women who were pregnant and those who had cancerous tumors were, however, omitted from the trial to minimize unneeded difficulties related to the operation. 

The Applied BiosystemSTM 7500 Fast Dx Real-Time Polymerase Chain Reaction (RT-PCR) was used to detect HPV genotypes 16, 18, 31, and 45 in the cervical swabs in accordance with the manufacturer's instructions (RT-PCR results were as follows: initial denaturation at 95°C for 2 mins, then 40 cycles of 95°C for 30 s, 65°C for 50 s, and 72°C for 10 s, followed by a terminal extension at 72°C for 5 mins). The kit used was HPV-HR genotyping TRUPCR kit (3B1290), which is an RT-PCR assay based on the oligonucleotide hydrolysis principle and allows higher specificity and sensitivity of E6/E7 region by primer and probes specific for HPV genotypes (16, 18, 31, and 45) and simultaneous genotyping of HPV-16 and 18.

The gathered data were examined for completeness before being entered. Simple frequencies and percentages were used to explain categorical epidemiological data, while means and standard deviation were used to express continuous or discrete data. To assess potential connections between categorical variables, Fisher's chi-squared test was applied. A statistically significant association is one with P < 0.05.

## Results

The clinical characteristics is described in Table 1 involving a total of 736 women between age group 21 to 60 that were included in the study.

**Table 1:** Demographic characteristics of patients

Characteristics	n = 736	Percentage (%)	Fisher's chi-squared test	p-value
Age in years				
21-30	215	29.2	260.0	0.001*
31-40	321	43.6		
41-50	132	17.9		
>50	68	9.3		
Marital status				
Never married	61	8.3	984.0	0.001*
Married	572	77.7		
Divorces	103	14.0		
Menopausal status				
Premenopausal	561	76.3	404.8	0.001*
Postmenopausal	175	23.7		
Education				
Primary and below	398	54.1	249.4	0.001*
Secondary education	115	15.6		
College and above	223	30.3		
Occupation				
House wife	284	38.5	361.8	0.001*
Employee	224	30.5		
Farmer	117	15.9		
Business	67	9.2		
Other	44	5.9		
Residence				
Urban	324	44.0	21.0	0.001*
Rural	412	56		

The knowledge of CC among patients is described in Table 2.

**Table 2:** Knowledge about CC CC: Cervical cancer; HPV: Human papillomavirus

	n = 736	Percentage (%)	Fisher’s chi-squared test	p-value
Heard about CC				
Yes	294	39.9	59.5	0.001*
No	442	60.1		
Heard about symptoms of CC				
Yes	148	20.1	526.0	0.001*
No	588	79.9		
Heard about HPV				
Yes	185	25.2	364.0	0.001*
No	551	74.8		
Vaccinated for HPV				
Yes	25	3.4	1278.7	0.001*
No	711	96.6		
Screened for HPV infection				
Yes	110	14.9	723.5	0.001*
No	626	85.1		

Table 3 summarizes the VIA CC screening positivity rate according to age group. Overall, 96 (13.0%) positivity rates and 640 (87.0%) negativity rates of VIA CC screening were found in participating women.

**Table 3:** VIA CC screening among participant women. VIA: Visual inspection with acetic acid; CC: Cervical cancer

Age in years	VIA CC screening					
	Positive		Negative		Fisher's χ 2	p-value
	No. of patients	%	No. of patients	%		
21-30	20	9.4	195	90.6	14.2	0.002*
31-40	39	12.2	282	87.8		
41-50	30	22.7	102	77.3		
>50	7	10.2	61	89.8		
Total	96	13.0	640	87.0	804.1	0.001*
Residence						
Urban	34	10.5	290	89.5	2.69	0.100
Rural	60	14.5	352	85.5		

Table 4 summaries, the genotyping of 16, 18, 31, and 45 of HPV was performed on women who were referred for standard CC screening during the study period, either separately or in combination based on age groups with RT-PCR.

**Table 4:** Prevalence of HPV genotypes hr-HPV: High-risk human papillomavirus

Age in years	Total (% of hr-HPV)	16	18	31	45	Combination 16, 17, 31, 45
21-30	18 (8.3%)	6 (33.3%)	2 (11.2%)	2 (11.2%)	1 (5.5%)	7 (38.8%)
31-40	19 (5.9%)	5 (26.3%)	2 (10.6%)	3 (15.7%)	2 (10.6)	7 (36.8)
41-50	14 (10.6%)	4 (28.5%)	1 (7.2%)	2 (14.3%)	2 (14.3%)	5 (35.7%)
>50	3 (4.4%)	1 (33.3%)	1 (33.3%)	0	0	1 (33.3%)
Total	54 (7.3%)	16 (29.6%)	6 (11.1%)	7 (12.9%)	5 (9.2%)	20 (37.0%)

Table 5 showed, the VIA positivity rate in women with high risk for the HPVs infection rate and the overall prevalence of hr-HPV infection and prevalence was provocatively more with VIA positivity rate with CC (79.6% in VIA positive and 20.3% in VIA negative).

**Table 5:** VIA positivity rate in women at hr-HPV infection and prevalence VIA: Visual inspection with acetic acid; CC: Cervical cancer; hr-HPV= High-risk human papillomavirus

VIA CC screening	Total % of hr-HPV	16	18	31	45	Combination 16, 17, 31 ,45
VIA positive N=96	43 (79.6%)	12 (27.9%)	4 (9.3%)	6 (13.9%)	3 (6.9%)	18 (41.8%)
VIA negative N=640	11 (20.3%)	4 (36.4%)	2 (18.2%)	1 (9.0%)	2 (18.2%)	2 (18.2%)

## Discussion

We prospectively assessed women samples from central India who had been there for a standard CC screening for genotypes of 16, 18, 31, and 45 hr-HPV using RT-PCR. These are the four most detected genotypes in CC, according to previous research [16]. The four genotypes of HPV were found at similar rates and did not vary significantly in single- and multiple-type infections between women in contrast to older women's cross-sectional studies [16-18]. The median age of the 736 female participants in this study ranged from 21 to 60. Due to the older mean age participants in part, the overall incidence of hr-HPV in the present study was less (7.3%) than in the other similar studies 12.6% and 11.7% [19-20]. Furthermore, it is equivalent to two previous Japanese studies on cotesting, where the prevalence of hr-HPV was 5.2% and 6.8%, respectively, in the studies with participants aged 20 to 69 [19,20].

The low average age of women with invasive CC attributed to HPV-16 or HPV-18 has been noted in other studies. HPV-45 is rare in women with normal cytology or low-grade lesions (0.4% and 3.7%, respectively) compared with HPV 16 (2.5% and 20%, respectively). However, it is consistently the third most common HPV type in invasive CC globally and in most of the regions [20]. The younger age of women presenting with invasive CCs that are positive for HPV types 16, 18, and 45 was consistent across study regions with substantially different uptake of screening [20]. This age variation is relevant for rationalization of the newly proposed screening policies and patient management protocols by use of specific genotype information [20,21].

The elimination of women who had visited family planning or gynecology clinics with symptoms of CC may account for the decreased HPV prevalence in the current study. In comparison to prior studies (13.1%, 14.1%, and 12.9% respectively), the current study's overall VIA positive rate of 13.0%, consisting of 10.5% in urban areas and 14.5% in rural areas, is comparable with the data from several Ethiopian regions [21-23]. The outcomes of this study suggest that combining VIA and HPV testing can help CC screening programs identify high-risk women early. The frequency of hr-HPV infection was alarmingly higher (79.6%) in women with CC who had VIA-positivity. To fully understand VIA positive in HPV-infected women, more study is necessary. Similar to a previous study the rate of cervical lesions and HPV infection in the present study was similar across all age groups, with a peak in the 40-9 years group [24]. However, elderly women are more prone to develop these infections and lesions [25]. The significant percentage of illiterate women in the rural area in the current study who had never known of CC suggests that there are lack of awareness and health-seeking behaviors in the area. Earlier research in Ethiopia revealed a similar result [26-28].

In this study, 16, 18, 31, and 45 genotypes of HPV had a prevalence rate of 29.6%, 11.1%, 12.9%, and 9.2%, each respectively, while the overall prevalence of hr-HPV was present in populations at 7.3% in individuals and 37.0% in combinations. HPV-16 genotype was identified as having a higher prevalence (29.6%). As studied by Torres-Ibarra et al, compared to HPV-18, 31, or 33, HPV-16 has been demonstrated to be a more accurate predictor of lesions' persistence [29].

Type-specific persistence of hr-HPV is the most well-known marker of risk of developing high-grade cervical precancers [28]. Long-term outcomes for general and public health are typically better when CC and precancers are detected early because women are less involved in interacting with medical services in low-resource regions [29]. However, more awareness programs are needed for a better understanding of CC and HPV testing.

## Conclusions

Individual hr-HPV genotype prevalence was shown to be lower than with combinations. The HPV-16 genotype was identified to have a higher prevalence than HPV-18, 31, and 45. An explanation for this might include early sexual development debut combined with low cervical screening attendance, particularly among women of reproductive age. Age-related HPV infections and cervical lesions were comparable across all age categories, however, are more common in older women, peaking in the age range of 40-9 years. Hr-HPV infection and prevalence were provocatively more with VIA-positivity rate with CC. A high percentage of participants were uneducated and from rural areas; this suggests that there is a lack of health-seeking behaviors and awareness in the area. The limited HPV testing and limited vaccination are also one more reason for providing high-risk populations.
